# Antibacterial Evaluation of Some Schiff Bases Derived from 2-Acetylpyridine and Their Metal Complexes

**DOI:** 10.3390/molecules17055952

**Published:** 2012-05-18

**Authors:** Nura Suleiman Gwaram, Hapipah Mohd Ali, Hamid Khaledi, Mahmood Ameen Abdulla, A. Hamid A. Hadi, Thong Kwai Lin, Chai Lay Ching, Cher Lin Ooi

**Affiliations:** 1 Chemistry Department, Faculty of Science, University of Malaya, Kuala Lumpur 50603, Malaysia; Email: hapipah@um.edu.my (H.M.A.); khaledi@gmail.com (H.K.); 2 Molecular Medicine Department, Faculty of Medicine, University of Malaya, Kuala Lumpur 50603, Malaysia; Email: ameen@um.edu.my; 3 Institute of Biological Science, Faculty of Science, University of Malaya, Kuala Lumpur 50603, Malaysia; Email: thongkl@um.edu.my (T.K.L.); lcchai@um.edu.my (C.L.C.)

**Keywords:** Schiff bases, metal complexes, x-ray crystallography, anti-bacteria

## Abstract

A series of Schiff bases derived from 2-acetylpyridne and their metal complexes were characterized by elemental analysis, NMR, FT-IR and UV-Vis spectral studies. The complexes were screened for anti-bacterial activity against Methicillin-resistant *Staphylococcus aureus* (MRSA), *Acinetobacter baumanni* (AC), *Klebsiella pneumonie* (KB) and *Pseudomonas aeruginosa* (PA) using the disc diffusion and micro broth dilution assays. Based on the overall results, the complexes showed the highest activities against MRSA while a weak antibacterial activity was observed against *A. baumanii* and *P. aeruginosa*.

## 1. Introduction

Schiff base ligands containing various donor atoms (like N, O, S, *etc*.) show broad biological activities and are of special interest due to variety of ways in which they can bond to metal ions. It is known that the existence of metal ions bonded to biologically active compounds may enhance their activities [1]. Schiff base metal complexes show great diversity in their varied biological activities as anticonvulsant [2], antifungal [3,4,5,6,7], anti-HIV [8], antiviral and anticancer [9] antimicrobial [10,11,12,13,14,15] and antibacterial [16] agents.

Nosocomial infections caused by multidrug resistant bacteria are an increasing medical problem worldwide, particularly among immunocompromised patients and those hospitalized in intensive care units. Both Gram positive and Gram negative bacteria have developed high level resistance to multiple classes of antibacterial agents. These include methicillin resistant *Staphylococcus aureus* (MRSA), *Pseudomonas aeruginosa*, *Acinetobacter baumannii*, *Escherichia coli* and vancomycin resistant enterococci (VRE) [17]. Few available drugs such as linezolid and some newer glycopeptides, and tigecycline are active against MRSA and VRE, but their success rates are variable [18]. As such, there is a need to explore other sources of effective antibacterial compounds to augment the limited choice of drugs for therapeutic treatment.

In this study, transition metal complexes of some newly synthesized *N,N′,N′*′-donor Schiff base ligands obtained from the reaction of 4-(2-aminoethyl)morpholine, 4-(2-aminoethyl)piperazine or *N,N*-dimethylethylenediamine with 2-acetylpyridine in the presence of Cl^−^, Br^−^, and SCN^−^ ions were examined for potential antimicrobial activities. Most of the synthesized compounds present a morpholine derivative which has been used as a building block for preparation of the antibiotic linezolid [19,20]. The morpholine ring is important for antimicrobial activity as proved by quantitative structure activity relationship (QSAR) [21]. The objective of the study was to determine the anti-bacterial activities for the transition metals complexes against methicillin-resistant *Staphylococcus aureus* (MRSA), *Acinetobacter baumanni* (*A. baumanni*), *Klebsiella Pneumonie* (*K. pneumonie*) and *Pseudomonas aeruginosa* (*P. aeruginosa*) by using the disc diffusion and micro broth dilution assays. 

## 2. Results and Discussion

**Scheme 1:**
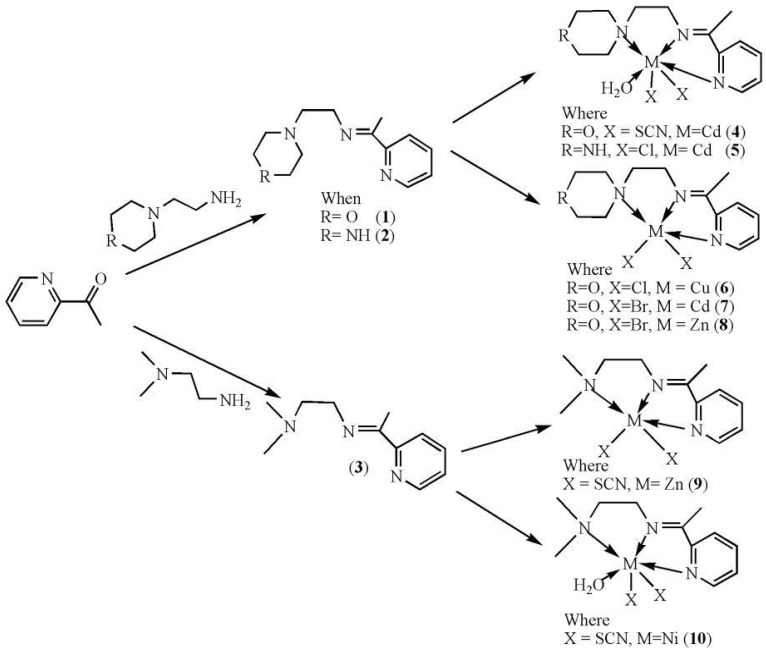
Reaction pathway for the coordinated complexes.

The reaction of 2-acetylpyridine with 4-(2-aminoethyl)morpholine, 4-(2-aminoethyl)piperazine or *N,N*-dimethylethylenediamine resulted in the formation of corresponding Schiff base ligands; 2-morpholino-*N*-(1-(pyridin-2-yl)ethylidene)ethanamine (**1**), 2-(piperazin-1-yl)-*N*-(1-(pyridin-2-yl)-ethylidene)ethanamine (**2**), *N1,N1*-dimethyl-*N2*-(1-(pyridin-2-yl)ethylidene)ethane-1,2-diamine (**3**). The prepared Schiff bases further reacted cadmium(II), copper(II), nickel(II) and zinc(II) ions in the presence of Cl^−^, Br^−^, SCN^−^ ions giving rise to different coordination complexes (Scheme 1). The compounds exhibited NMR, IR and UV-Visible spectra consistent with the proposed structures which allowed the synthesized complexes to be recognized as aqua{dithiocyanato-(2-morpholino-*N*-[1-(2-pyridylethylidene]ethanamine *N,N′,N′*′}cadmium(II) (**4**), aqua{dichloridopiperazin-4-ium1-(2-pyridyl) ethylidene]ethanamine *N,N′,N′*′}cadmium (**5**), chlorido(2-{1-[(2-morpholinoethyl)imino]ethyl}-phenolato *N,N′,N′*′)copper(II) (**6**), dibromido{2-morpholino-*N*-[1-(2-pyridyl)ethylidene)ethanamine *N,N′,N′*′}cadmium (**7**), dibromido{2- *N,N′,N′*′}bis(thiocyanato) nickel(II) (**10**), respectively. In this context, it would be worthy morpholino-*N*-[1-(2-pyridyl)ethylidene] ethanamine *N,N′,N′*′} zinc (**8**), *N,N*-dimethyl-*N’*-[1-(2-pyridyl)ethylidene]ethane-1,2-diamine-^3^*N,N′,N′*′}bis(thiocyanate) zinc(II) (**9**), and aqua{2-morpholino-*N*-[1-(2-pyridyl)ethylidene]ethanamine to compare the structures of some earlier prepared complexes with the present ones. There are two reported complexes of nickel(II) perchlorate with the similar donor-Schiff base ligand [22].

### 2.1. Infrared Spectra

The IR spectra of the compounds were recorded in the 4000–400 cm^−1^ range. The characteristic IR stretching frequencies along with their proposed assignments are summarized in the Experimental section. There are similarities in the IR spectra of the ligands and their corresponding metal complexes to each other, except for some slight variations in the shifts and intensities caused by different metal(II) ions. However, there were some significant differences between the metal(II) halides complexes and those of the thiocyanate complexes, as expected. The IR spectra of all the compounds possess very strong characteristic absorption bands in the 1,585–1,656 cm^−1^ region which are attributed to the C=N stretching vibration of the Schiff base functional group [11,23,24]. The hydrated complexes (**4**, **5** and **10**) exhibited an IR band at approximately 650–700 cm^−1^ range due to ν(H_2_O), suggesting the coordination of water molecules [25,26]. The characteristic sharp and distinct absorption bands for N=C=S appear in the 2,030–2,120 cm^−1^ region for compounds **4**, **9** and **10** [27,28]. All complexes showed M-N bands at a lower wavelength in the range of 454–556 cm^−1^ [29,30,31,32].

### 2.2. Electronic Spectra

The electronic spectra for all the compounds were obtained in DMSO solutions and showed absorption bands in three distinct regions. The first region ranging from 200 to approximately 229 nm, is characteristic for the electronic inter-ligand π→π* transitions [33], while the second characteristic wavelength in the region of 280 nm to approximately 350 nm is the second inter ligand n→π transition [34]. The third distinct region ranging from 407 nm to approximately 500 nm is the characteristic for the ligand to metal charge transfer (LMCT) from the nitrogen atom to the transition metal centre [35]. For the nickel(II) and copper(II) ions, bands were observed between 600 and 750 nm which can be attributed to d–d* transitions of the metal ions respectively [36].

### 2.3. ^1^H- and ^13^C-NMR-NMR Spectra

The signals for the aromatic ring protons appear in the region of 7.80–8.80 [37]. While the other singlet peaks appeared in the region of 1.80–2.65 ppm which were attributed to CH_3_ indicating the methyl on the carbonyl group [37]. Also in the piperazine compound **5** additional signals was observed at 2.30 ppm which was assigned to (NH–). In the ^13^C-NMR spectra of the metal complexes, the signals in the range from 160.00–170.00 ppm were assigned to azomethine carbon atoms (C=N) [32]. Aromatic ring carbon atoms were determined in the range of 120.00–150.00 ppm [37]. Additional signals were observed at 21.98 ppm and 22.75 ppm, which were assigned to the carbon atom of (N*C*S) for compounds **4** and **9**, respectively.

### 2.4. X-Ray Crystal and Molecular Structure

**Figure 1:**
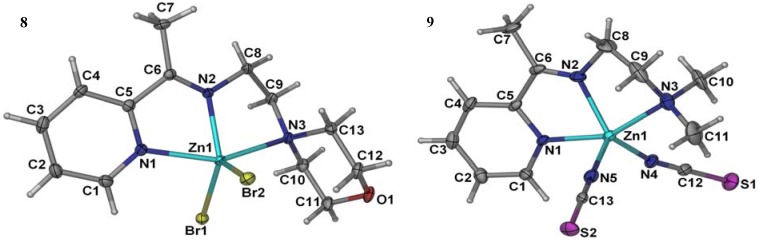
The molecular X-ray structure of **8** and **9** showing the atom labeling scheme (50% probability ellipsoids). Hydrogen atoms are drawn as spheres of arbitrary radius.

Compound **8** was obtained upon the reaction of 2-morpholino-*N*-[1-(2-pyridyl)ethylidene]-ethanamine with Zn(II) ion in the presence of potassium bromide. Similar to the structure of the analogous ZnCl_2_ complex [38], the metal center is five-coordinated by the *N,N′,N′′-*tridentate Schiff base ligand and two halogen atoms (Figure 1). 

The geometry of the complexes can be determined by using the index τ = (β-α)/60, where β is the largest angle and α is the second one around the metal center. For an ideal square-pyramid τ is 0, while it is 1 in a perfect trigonal-bipyramid [39]. The τ value in the present structure is calculated to be 0.30, indicative of a distorted square-pyramidal geometry. The Zn-Br and Zn-*N* bond lengths in the complex are in agreement with the values reported in the literature [40,41]. The Zn(II) ion in the compound, is five-coordinated by the *N,N′,N′′-*tridentate Schiff base ligand and two Br atoms in a distorted square-pyramidal geometry. The three Zn-N bond lengths as apparent from (Table 1) are given as Zn(1)-*N*(1) 2.243(3) Å, Zn(1)-N(2) 2.076(2) Å, Zn(1)-N(3) 2.331(2) Å, and two Zn-Br bond lengths are Zn(1)-Br(1) 2.4036(4) (18) Å, Zn(1)-Br(2) 2.4172(4) Å. In the crystal, intermolecular C---H...Br hydrogen bonds link the adjacent molecules into infinite chains along the crystallographic axis. An intramolecular C---H...Br interaction is also observed. Table 2 summarizes the crystal data for compounds **8** and **9** zinc(II) complexes.

**Table 1:** Selected bond lengths [Ǻ] and Selected bond angles [°] for compounds **8** and **9**.

8				9			
Zn1	Br1	2.4036(4)		Zn1	N1	2.1752(18)	
Zn1	Br2	2.4172(4)		Zn1	N2	2.0752(18)	
Zn1	N1	2.243(3)		Zn1	N3	2.217(2)	
Zn1	N2	2.076(2)		Zn1	N4	1.9681(18)	
Zn1	N3	2.331(2)		Zn1	N5	1.9698(19)	
Br1	Zn1	Br2	116.905(15)	N1	Zn1	N3	154.18(7)
N1	Zn1	Br1	92.48(6)	N2	Zn1	N1	75.30(7)
N1	Zn1	Br2	95.00(6)	N2	Zn1	N3	79.64(8)
N1	Zn1	N3	151.50(8)	N4	Zn1	N1	97.48(7)
N2	Zn1	Br1	133.79(6)	N4	Zn1	N2	133.47(8)
N2	Zn1	Br2	108.28(6)	N4	Zn1	N3	95.22(7)
N2	Zn1	N1	73.92(9)	N4	Zn1	N5	113.13(8)
N2	Zn1	N3	79.37(9)	N5	Zn1	N1	96.56(7)
N3	Zn1	Br1	99.01(6)	N5	Zn1	N2	113.35(8)
N3	Zn1	Br2	102.71(5)	N5	Zn1	N3	98.97(8)
C1	N1	Zn1	128.0(2)	C1	N1	Zn1	126.71(14)
C5	N1	Zn1	113.25(18)	C5	N1	Zn1	114.26(14)
C6	N2	Zn1	120.85(19)	C6	N2	Zn1	120.19(15)
C8	N2	Zn1	115.86(19)	C8	N2	Zn1	114.51(17)
C9	N3	Zn1	100.28(17)	C9	N3	Zn1	100.2(2)
C13	N3	Zn1	115.48(17)	C10	N3	Zn1	112.0(2)
C6	N2	C8	122.7(2)	C11	N3	Zn1	107.3(3)
C8	N2	Zn1	115.86(19)	C9'	N3	Zn1	104.0(2)
C9	N3	C13	109.5(2)	C11	N3	Zn1	112.3(2)
C1	N1	C5	118.7(3)	C12	N4	Zn1	162.3(2)
C10	N3	C13	107.5(2)	C13	N5	Zn1	175.5(2)

**Table 2:** Crystal data for compounds **8** and **9**.

	8	9
Empirical formula	C_13_H_19_Br_2_N_3_OZn	C_13_H_17_N_5_S_2_Zn
Formula weight	458.50	372.81
Temperature/K	100(2)	100(2)
Crystal system	monoclinic	monoclinic
Space group	P2_1_/n	*P2_1_/n *
a/Å	9.8290(2)	13.7663(2)
b/Å	14.0218(2)	9.4949(2)
c/Å	12.1371(2)	13.8089(2)
β/°	106.9180(10)	109.9460(10)
Volume/Å^3^	1600.35(5)	1696.68(5)
Z	4	4
ρ_calc_mg/mm^3^	1.903	1.459
m/mm^−1^	6.527	1.693
F(000)	904	768
Crystal size/mm^3^	0.33 × 0.25 × 0.11	0.35 × 0.31 × 0.19
2Θ range for data collection	4.56 to 54°	5.14 to 54°
Reflections collected	12535	15059
Independent reflections	3496[*R(int)* = 0.0351]	3707[*R(int)* = 0.0232]
Data/restraints/parameters	3496/0/182	3707/14/225
Goodness-of-fit on F^2^	1.028	1.015
Final R indexes [I >= 2σ (I)]	*R_1_* = 0.0271, wR_2_ = 0.0595	*R_1_* = 0.0296, *wR_2_* = 0.0714
Final R indexes [all data]	*R_1_* = 0.0360, wR_2_ = 0.0624	*R_1_* = 0.0402, *wR_2_* = 0.0772
Largest diff. peak/hole/e Å^−3^	0.679/−0.466	0.455/−0.428

Compound **9** is a square-pyramidal zinc(II) complex in which the metal center is coordinated by the *N,N′,N′′-*tridentate Schiff base and the *N* atoms of two thiocyanate ligands (Figure 1). The CH_2_*N*(CH_3_)_2_ fragment is disordered over two sets of sites in a 0.529 (4):0.471 (4) ratio. Like the structure of the zinc(II) thiocyanate complexes of similar Schiff bases [42,43], the present structure is a mononuclear square-pyramidal metal complex, the τ value being 0.35. In contrast, the cadmium(II) thiocyanate complex of the same Schiff base [44], showed a polymeric structure wherein the metal centers are linked by the N:S-bridging thiocyanate ligands. In the crystal structure of the title compound, C---H...S interactions connect a pair of molecules, related by symmetry −x+1, −y, −z, around a center of inversion. The zinc atom is bonded to three donor nitrogen atoms of the ligand and two thiocyanates both bonded through the nitrogen atoms. The five Zn-*N* bond lengths as apparent from (Table 1) are given as Zn(1)-N(1) 2.1752(18) Å, Zn(1)-N(2) 2.0752(18) Å, Zn(1)-N(3) 2.217(2) Å, Zn(1)-N(4) 1.9681(18) Å, Zn(1)-N(5) 1.9698(19) Å.

### 2.5. Antibacterial Activity Results

**Figure 2:**
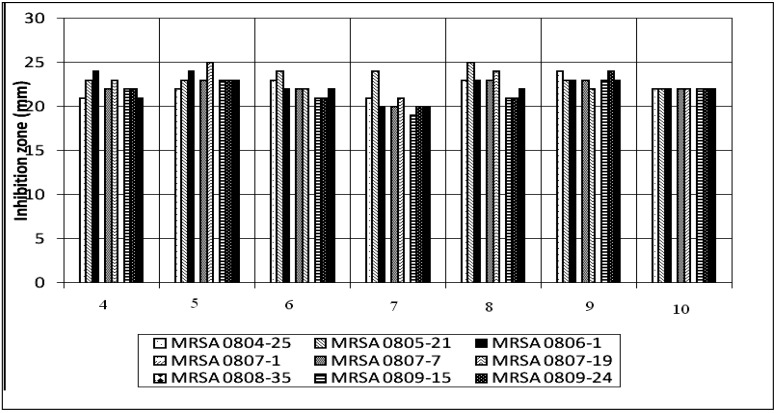
Histogram for the 10 MRSA clinical strains of Schiff-base complexes.

A total of ten Schiff-bases compounds were synthesized and screened for potential antibacterial activity against the emerging multi-drug resistance nosocomial bacterial pathogens in hospital settings, namely, methicillin-resistant *Staphylococcus aureus*, *A. baumanii*, *K. pneumonia* and *P. aeroginosa*. In the first level antibacterial screening with the disc diffusion assay, all compounds under study were tested against eight clinical strains, two from each bacterial species. Inhibition zones were observed in the Schiff base compounds. However, no inhibition zone was observed in any of the compounds tested against *K. pneumonia* (Table 3). All seven transition metal-bonded Schiff-base compounds were strongly inhibitive against MRSA, while they showed weak antibacterial activity against *A. baumanii* and *P. aeroginosa* in the disc diffusion test (Table 3). This higher antimicrobial activity of the metal complexes **4**–**10**, compared with that of Schiff base ligands **1**–**3**, is perhaps due to the change in structure as a result of coordination, as chelating tends to make metal complexes act as more powerful and potent bacteriostatic agents, thus inhibiting the growth of the microorganisms [45,46]. These seven compounds were then analyzed further for the MIC and MBC value using broth micro-dilution assay (Table 4). The result from broth micro-dilution assay was in total analogue to the disk diffusion screening. The test showed that, all of the seven compounds were most active against MRSA, with MIC value ranged from 0.7–2.9 µg/mL; MBC value ranged from 2.9–46.9 µg/mL (Table 4). Both *A. baumanii* and *P. aeroginosa* were resistant to the tested compounds at concentration lower than 93.8 and 187.0 µg/mL, respectively (Table 4). Therefore, it was concluded that these seven compounds were highly inhibitive only against the Gram positive bacterium MRSA. Further antibacterial tests were conducted against eight clinical strains of multi-drug resistant MRSA (Table 5) showing varying responses towards the seven selected Schiff-based compounds in the disc diffusion assay (Figure 2), however, they demonstrated twenty percent of resistance rate (two strains out of ten were resistant to the seven tested compounds). The broth micro-dilution assay resulted in similar findings, in which all, but MRSA 0807-1 and MRSA 0808-35, were inhibited at MIC ranging from “1.5 to 11.7” µg/mL. Both, MRSA 0807-1 and MRSA 0808-35, were only inhibited at MIC ranging from “209.7 to 419.4” µg/mL. (Table 6) presented the antibiogram of ten clinical strains of MRSA used in this antibacterial analysis with some known antibacterial drugs in which MRSA either showed resistant (R), susceptible (S) or intermediate resistant (I) towards them.

**Table 3:** First level antibacterial screening Disc diffusion assay.

Compound	MRSA 0804-25	MRSA 0807-7	AC 0612-7	AC 0903-21	KB 71	KB 83	PA 45	PA 104
1	6	6	0	0	0	0	5	6
2	5	5	0	0	0	0	5	5
3	0	0	0	0	0	0	0	0
4	21	22	7	7	0	0	8	8
5	24	23	9	8	0	0	11	19
6	22	23	9	8	0	0	10	10
7	23	22	10	10	0	0	8	8
8	21	20	10	9	0	0	9	9
9	23	23	9	8	0	0	10	9
10	22	22	7	7	0	0	10	9
Control (DMSO)	0	0	0	0	0	0	0	0

**Table 4:** Second level antibacterial screening.

Minimum inhibitory concentration (MIC; µg/mL)/minimum bactericidal concentration (MBC; µg/mL)								
	MRSA 0804-25	MRSA 0807-7	AC 0612-7	AC 0903-21	KB 71	KB 83	PA 45	PA 104
1-3	n.d.	n.d.	n.d.	n.d.	n.d.	n.d.	n.d.	n.d.
4	0.7/2.9	1.5/2.9	93.8/750	93.8/750	375/750	375/750	187.5/750	187.5/750
5	0.7/2.9	0.7/2.9	93.8/750	93.8/750	187.5/750	187.5/750	187.5/750	187.5/750
6	1.5/5.9	1.5/5.9	93.8/375	93.8/375	375/750	375/750	187.5/750	187.5/750
7	1.5/5.9	1.5/5.9	93.8/375	93.8/375	187.5/750	187.5/750	187.5/750	187.5/750
8	1.5/5.9	1.5/2.9	93.8/750	93.8/750	375/750	375/750	187.5/750	187.5/750
9	1.5/5.9	1.5/23.4	187.5/750	187.5/750	375/750	375/750	187.5/750	187.5/750
10	2.9/46.9	2.9/46.9	187.5/750	187.5/750	375/750	375/750	187.5/750	187.5/750
DMSO	375/>375	375/>375	375/>375	375/>375	375/>375	375/>375	375/>375	375/>375

n.d. = not determined.

**Table 5:** Varying responses of the eight MRSA clinical strains towards the seven selected Schiff-base complexes in the disc diffusion assay.

Compounds	MRSA 0805-21	MRSA 0806-1	MRSA 0807-1	MRSA 0807-19	MRSA 0808-35	MRSA 0809-15	MRSA 0809-25	MRSA 0809-38
**4**	23	24	0	23	0	22	22	21
**5**	23	24	0	25	0	23	23	23
**6**	24	22	0	22	0	21	21	22
**7**	24	20	0	21	0	19	20	20
**8**	25	23	0	24	0	21	21	22
**9**	23	23	0	22	0	23	24	23
**10**	22	22	0	22	0	22	22	22
**(DMSO)**	0	0	0	0	0	0	0	0

**Table 6:** Antibiogram of the ten clinical strains of MRSA used in the antibacterial analysis.

Strain No	E	CN	LZD	MUP	RD	DA	TEC	CIP	NET	TE	VA	FD	OX	SXT
MRSA 0805-21	R	R	S	S	S	S	S	R	R	R	S	S	R	R
MRSA 0806-1	R	R	S	S	S	S	S	R	R	R	S	S	R	R
MRSA 0807-1	R	R	S	S	S	S	S	R	R	R	S	S	R	R
MRSA 0807-19	R	R	S	S	S	S	S	R	I	S	S	S	R	S
MRSA 0808-35	R	R	S	S	S	S	S	I	I	R	S	S	R	R
MRSA 0809-15	R	R	S	S	S	S	S	R	R	R	S	S	R	R
MRSA 0809-24	R	R	S	S	S	S	S	R	R	R	S	S	R	R
MRSA 0809-38	R	R	S	S	S	S	S	R	R	R	S	S	R	S
MRSA 0807-7	R	R	S	S	S	S	S	R	I	R	S	S	R	R
MRSA 0804-25	R	R	S	S	S	S	S	R	S	S	S	S	R	S

E = erythromycin; GN = gentamycin; LZD-linozolid acid; MUP = muciprocin; RD- rifampicin; DA = clindamycin; TEC = teicoplanin; CIP = ciproflozaxin; NET = netilmycin; TE = tetracycline; VA = vancomycin; FD = fusicidic acid; OX = oxacilin; SXT = Trimethoprim-sulfamethaxazole; R = resistant; S = sensitive; I = intermediate resistant.

**Figure 3:**
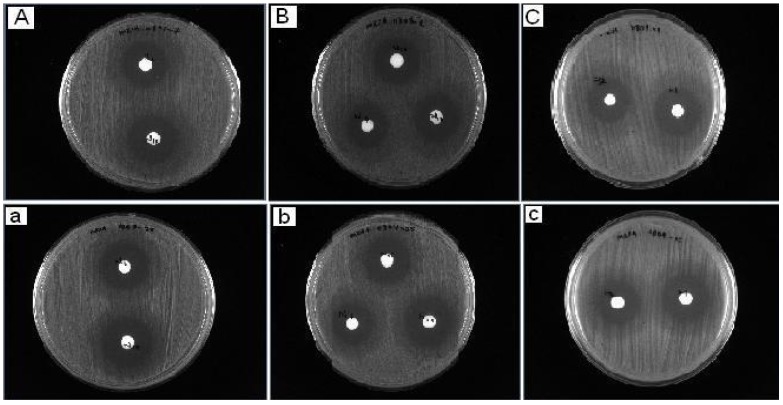
Photograph showing the antibacterial (MRSA 0804-25-A,B,C) and (MRSA 0807-7-a,b,c) screening.

## 3. Experimental

### General

Melting points were determined using a MEL-TEMP II melting point instrument and were not corrected. Microanalyses were carried out on a Perkin-Elmer 2400 elemental analyzer. ^1^H-NMR and ^13^C-NMR spectra were determined with a Lambda JEOL 400 MHz FT-NMR (^1^H: 400 MHz and ^13^C: 100.4 MHz) spectrometer. Chemical shifts are given in δ values (ppm) using TMS as the internal standard. The IR spectra were taken with a Perkin-Elmer RX1 FT-IR spectrophotometer. 

*2-Morpholino-N-(1-(pyridin-2-yl)ethylidene)ethanamine *(**1**): A mixture of 4-(2-aminoethyl)-morpholine (0.65 g, 5 mmol) and 2-acetylpyridine (0.61 g, 5 mmol) in ethanol (50 mL) was refluxed for 2 h followed by addition of few drops of glacial acetic acid. The obtained orange oil was solidified upon standing for 12 h at 55 °C in an oven.


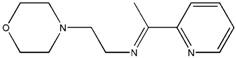


The solid product was recrystallized in methanol. Yield: 61.5%. Molecular formula: C_13_H_19_N_3_O. Analysis Calculated: C, 66.92; H, 8.21; N, 18.01. Found: C, 67.02; H, 8.52; N, 17.99. IR (ATR cm^−1^) 2972.40 n(C-H), 1690.00, 1642.08 n(C=N), 1588.74, 1568.03 n(C=N)pyr, 1450.89 n(C-C), 1113.20 n(C-N), 1089.21 n(O-C). UV-Vis (DMSO), 281.00 (n→π*); 224.00 (π→π*). ^1^H-NMR (DMSO-*d_6_*), 8.69–7.95 (4H, CH-Ar), 3.79–3.837 (CH_2_-morph), 2.78–2.58 (NCH_2_-CH_2_N), 1.74 (CH_3_-). ^13^C-NMR (DMSO-*d_6_*). 164.53 [1C, δ(C=N)], 144.45 δ(C), 142.62 δ(CH), 136.40 δ(CH), 123.96 δ(CH), [5C, δ(Ar-pyr)], 61.23 [2C, δ(2CH_2_)], 52.08, 49.67 [2C, δ(2CH_2_)], 44.57 [C, δ(CH_2_)], 31.82 [2C, δ(CH_2_)], 14.52 [C, δ(CH_3_)].

*2-(Piperazin-1-yl)-N-(1-(pyridin-2-yl)ethylidene)ethanamine *(**2**): A mixture of 4-(2-aminoethyl)-piperazine (0.65 g, 5 mmol) and 2-acetylpyridine (0.61 g, 5 mmol) in ethanol (50 mL) was refluxed for three hours followed by addition of few drops of glacial acetic acid.


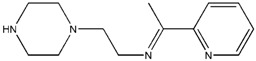


The same procedure as for **1** was applied as the prototype in the isolation of other Schiff bases. Yield: 40.3%. Molecular formula: C_13_H_20_N_4_. Analysis Calculated: C, 67.21; H, 8.68; N, 24.12. Found: C, 68.19; H, 8.89; N, 24.48. IR (ATR cm^−1^) 3308.37 n(N-H), 2945.30 n(C-H), 1640.05 n(C=N), 1586.63 n(C=N)pyr, 1464.94, 1436.55 n(C-C), 1132.42 n(C-N). UV-Vis (DMSO) 281.00 (n→π*); 224.00 (π→π*). ^1^H-NMR (DMSO-*d_6_*) 8.30–7.85 (4H, CH-Ar), 3.98–3.69 (CH_2_-Pip.), 2.9 (NH-) 2.75–2.55 (NCH_2_-CH_2_N), 1.21 (CH_3_-). ^13^C-NMR (DMSO-*d_6_*) 168.43 [1C, δ(C=N)], 148.47 δ(C), 140.44 δ(CH), 127.93 δ(CH), 123.79 δ(CH), [5C, δ(Ar-pyr)], 51.72 [2C, δ(2CH_2_)], 44.19 [2C, δ(2CH_2_)], 42.04 [2C, δ(CH_2_)], 15.79 [1C, δ(CH_3_)].

*N1,N1-Dimethyl-N2-(1-(pyridin-2-yl)ethylidene)ethane-1,2-diamine *(**3**): An accurately measured amount of 2-acetylpyridine (0.61 g, 5 mmol) and *N*,*N*-dimethylethyldiamine (0.44 g, 5 mmol) in ethanol (25 mL) was refluxed for three hours.


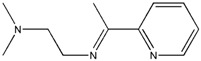


Yield: 54%. Molecular formula: C_11_H_17_N_3_ (191.27). Analysis Calculated: C, 69.87; H, 8.80; N, 13.58. Found: C, 68.48; H, 8.98; N, 14.98. IR (ATR cm^−1^) 2925.46 n(C-H) 1639.70 n(C=N) 1566.34 n(C=N)pyr, 1464.83, 1435.25 n(C-C), 1153.71, 1103.38 n(C-N). UV-Vis (DMSO) 321 (n→π*); 233 (π→π*).^1^H-NMR (DMSO-*d_6_*) 8.28–7.87 (CH_2_-Ar), 2.76–2.68 (NCH_2_-CH_2_N), 1.22 (CH_3_-), 1.9 (6H, CH_3_-N). ^13^C-NMR (DMSO-*d_6_*) 166.59 [1C, δ(C=N)], 147.58 δ(C), 145.83 δ(CH), 139.56 δ(CH), 126.82 δ(CH), [5C, δ(Ar-pyr)], 54.43 [2C, δ(2CH_2_)], 43.44. 43.29 [2C, δ(2CH_3_)], 14.69 [1C, δ(CH_3_)].

*Aqua{Dithiocyanato-(2-morpholino-N-[1-(2-pyridylethylidene]ethanamine N,N',N''}cadmium(II) *(**4**): A mixture of 2-acetylpyridine (0.20 g, 1.65 mmol) and 4-(2-aminoethyl)morpholine (0.21 g, 1.65 mmol) in ethanol (20 mL) was refluxed for 2 h followed by addition of a solution of cadmium(II) acetate dihydrate (0.44 g, 1.65 mmol) and sodium thiocyanate (0.268 g, 3.30 mmol) in a minimum amount of water. The mixture was refluxed for 2–3 h resulting in the formation of small amount of precipitate. More precipitate was obtained by removal of some solvent. 


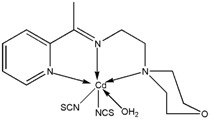


The product was collected by filtration, washed several times with ethanol until a white colored compound is obtained. It was re-crystallized from the same solvent (ethanol), filtered to remove the suspended impurities. Yield = 75%. Melting point >400 °C. Analysis Calculated: [C, 39.09; H, 4.37; N, 18.23; S, 13.91]. Found: [C, 39.29; H, 4.17; N, 18.13; S, 13.61]. IR (ATR cm^−1^) 2981.09 n(C-H), 2157.91, 2083, n(N=C=S), 1651.16 n(C=N), 1438.25 n(C-C), 1107.15 n(C-N), 659.88 n(OH_2_) 565.42 n(M-N). UV-Vis (DMSO) 407 (LMCT), 285 (n→π*); 234 (π→π*). ^1^H-NMR (DMSO-*d_6_*) 8.80 [s, 1H, δ(Ar-H)pyr], 8.29–8.24 [m, 2H, δ(Ar-H)pyr], 7.87–7.85 [t, 1H, δ(Ar-H)pyr], 3.80, 3.76 [d, 4H, δ(2CH_2_)], 3.69 [s, 4H, δ(2CH_2_)], 2.79–2.77 [t, 4H, δ(N-CH2)], 2.69 [s, 3H, δ(CH_3_)]. ^13^C-NMR (DMSO-*d_6_*) 165.80 [1C, δ(C=N)], 149.24 δ(C), 140.55 δ(CH), 132.31 δ(CH), 127.68 δ(CH), 124.29 δ(CH) [5C, δ(Ar-pyr)], 64.76, 57.88 [4C, δ(2CH_2_) morph], 53.44, 44.36 [2C, δ(2CH_2_)], 21.98 [δ(SCN)], 15.77 [1C, δ(CH_3_)].

*Aqua{Dichlorido Piperazin-4-ium1-(2-pyridyl)ethylidene]ethanamine N,N′,N′′}cadmium *(**5**): A mixture of 2-acetylpyridine (0.61 g, 5 mmol) and 4-(2-aminoethyl)piperazine (0.65 g, 5 mmol) in ethanol (50 mL) was refluxed for 2 h followed by addition of a solution of cadmium(II) chloride (0.92 g, 5 mmol) in a minimum amount of water was added. The mixture was refluxed for 2–3 h resulting in the formation of white precipitate.


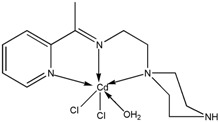


More precipitate was obtained by removal of some solvent. Analysis Calculated: C, 23.61; H, 3.55; N, 10.14. Found: C, 23.65; H, 3.75; N, 10.24. IR (ATR cm^−1^) 3469.07 n(N-H), 2953.13 n(C-H), 1654.02 n(C=N), 1438.11 n(C-C), 1121.21 n(C-N), 652.14 n(OH_2_) 557.10 n(M-N). UV-Vis (DMSO) 497 (LMCT), 281 (n→π*); 229 (π→π*). ^1^H-NMR (DMSO-*d_6_*) 8.73–7.83 [m, 4H, δ(Ar-H)pyr], 3.92, 3.81 [d, 4H, δ(2CH_2_)], 3.3–3.0 [t, 4H, δ(2CH_2_)], 2.82–2.80 [2H, δ(N-CH2)], 2.76 [2H, δ(NH-)], 2.38–2.36 [t, 2H, δ(CH_2_=)], 2.30 [sbr, 1H, δ(NH)] 2.23 [s, 3H, δ(CH_3_)]. ^13^C-NMR (DMSO-*d_6_*) 166.938 [1C, δ(C=N)], 157.942, 146.101, 137.967, 131.059, 124.085 [5C, δ(Ar-pyr)] 108.095, 106.975 [δ(2CH_2_)], 69.65 [2C, δ(2CH_2_)], 40.67 [1C, δ(CH_2_)], 46.00 [1C, δ(CH_2_)], 18.95 [1C, δ(CH_3_)].

*Chlorido(2-{1-[(2-morpholinoethyl)imino]ethyl}phenolato N,N′,N′′)copper(II) *(**6**): A mixture of 2-acetylpyridine (0.20 g, 1.65 mmol) and 4-(2-aminoethyl) morpholine (0.21 g, 1.65 mmol) in ethanol (20 mL) was refluxed. After 2 h a solution of copper (II) chloride dihydrate (0.28 g, 1.65 mmol) in a minimum amount of ethanol was added. The mixture was refluxed for 2–3 h resulting in the formation of a small precipitate. More precipitate was obtained by removal of some solvent.


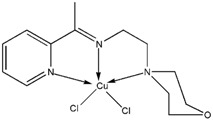


The product was collected by filtration, washed several times with ethanol until a green colored compound is obtained. It was re-crystallized from the same solvent (ethanol), filtered to remove the suspended impurities. Yield = 77%. Melting point 375–380 °C. Analysis Calculated: [C, 42.46; H, 5.21; N, 11.43]. Found: [C, 42.19; H, 5.11; N, 11.39]. IR (ATR cm^−1^) 2949.37 n(C-H), 1654.51 n(C=N), 1443.03 n(C-C), 1144.39 n(C-N), 577.38 n(M-N). UV-Vis (DMSO) 645.50 (d→d*); 337.00 (LMCT); 309.00 (n→π*); 223.00, 219.00 (π→π*).

*Dibromido{2-morpholino-N-[1-(2-pyridyl ethylidene ethanamine N,N′,N′′}cadmium *(**7**): A mixture of 4-(2-aminoethyl)morpholine (0.65 g, 5 mmol) and 2-acetylpyridine (0.61 g, 5 mmol) in ethanol (50 mL) was refluxed for 2 h followed by addition of a solution of cadmium(II) acetate dihydrate (0.44 g, 1.65 mmol) and potassium bromide (0.196 g, 1.65 mmol) in a minimum amount of water was added. The mixture was refluxed for 2–3 h resulting in the formation of a white precipitate. More precipitate was obtained by removal of some solvent.


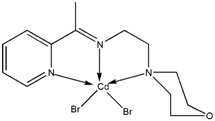


The product was collected by filtration, washed several times with ethanol until a white colored compound is obtained. It was re-crystallized from the same solvent (ethanol), filtered to remove the suspended impurities. Yield = 81%. Melting point >400 °C. Analysis Calculated: C, 37.48; H, 4.60; N, 10.09. Found: C, 37.28; H, 4.45; N, 10.19. IR (ATR cm^−1^) 296202 n(C-H), 1650.04 n(C=N), 1458.80 n(C-C), 1115.11 n(C-N), 556.01 n(M-N). UV-Vis (DMSO) 398 (LMCT), 279 (n→π*); 229 (π→π*). ^1^H-NMR (DMSO-*d_6_*) 8.69–8.68 [d, 1H, δ(Ar-H)pyr], 8.29–8.24 [m, 2H, δ(Ar-H)pyr], 7.85 [s, 1H, δ(Ar-H)pyr], 3.93, 3.80 [d, 4H, δ(2CH_2_)], 3.73–3.71 [t, 2H, δ(N-CH2)], 2.81, 2.80, 2.80, 2.72, 2.70 [m, 6H, δ(3CH_2_) morph], 2.55 [s, 3H, δ(CH_3_)]. ^13^C-NMR (DMSO-*d_6_*) 166.90 [1C, δ(C=N)], 149.13 δ(C), 147.64 δ(CH), 140.55 δ(CH), 127.72 δ(CH), 124.42 δ(CH), [5C, δ(Ar-pyr)], 64.65 [2C, δ(2CH_2_)], 53.49 [2C, δ(2CH_2_)], 36.00 [1C, δ(CH_2_)], 30.67 [1C, δ(CH_2_)], 15.95 [1C, δ(CH_3_)].

*Dibromido {2-morpholino-N-[1-(2-pyridyl) ethylidene] ethanamine N,N′,N′′}zinc *(**8**): A mixture of 2-acetylpyridine (0.20 g, 1.65 mmol) and 4-(2-aminoethyl) morpholine (0.21 g, 1.65 mmol) in ethanol (20 mL) was refluxed. After 2 h a solution of zinc (II) acetate dihydrate (0.36 g, 1.65 mmol) and potassium bromide (0.196 g, 1.65 mmol) in a minimum amount of water. The mixture was refluxed for 2–3 h resulting in the formation of a small amount of precipitate. More precipitate was obtained by removal of some solvent.


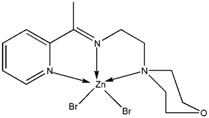


The product was collected by filtration, washed several times with ethanol until a milky colored compound is obtained. It was re-crystallized from the same solvent (ethanol), filtered to remove the suspended impurities and a single crystal was obtained suitable for x-ray analysis. Yield = 85%. Melting point >400 °C. Analysis Calculated: C, 30.89; H, 3.79; N, 8.31. Found: C, 30.79; H, 3.49; N, 8.28. IR (ATR cm^−1^) 2959.01 n(C-H), 1649.00 n(C=N), 1439.00 n(C-C), 1114.11 n(C-N), 561.12 n(M-N). UV-Vis (DMSO) 498 (LMCT), 289 (n→π*); 229 (π→π*). ^1^H-NMR (DMSO-*d_6_*) 8.78, 8.77 [d, 1H, δ(Ar-H)pyr], 8.30–8.25 [m, 2H, δ(Ar-H)pyr], 7.89–7.87 [t, 1H, δ(Ar-H)pyr], 3.89 [s, 4H, δ(2CH_2_)], 3.85–3.83 [t, 2H, δ(CH_2_)], 2.91–2.90 [t, 6H, δ(N-CH2)], 2.62 [s, 3H, δ(CH_3_)]. ^13^C-NMR (DMSO-*d_6_*) [1C, δ(C=N)], 168.38 δ(C), 146.49 δ(CH), 140.65 δ(CH), 128.03 δ(CH), 124.05 δ(CH) [5C, δ(Ar-pyr)], 62.88 [2C, δ(2CH_2_)], 54.55 [2C, δ(2CH_2_)], 52.09 [1C, δ(CH_2_)], 44.12 [1C, δ(CH_2_)], 15.86 [1C, δ(CH_3_)].

*N,N-Dimethyl-N'-[1-(2-pyridyl)ethylidene]ethane-1,2-diamine-^3^N,N′,N′′}bis(thiocyanate)zinc(II) *(**9**): A mixture of 2-acetylpyridine (0.61 g, 5 mmol) and *N*,*N*-dimethylethyldiamine (0.44 g, 5 mmol) in ethanol (50 mL) was refluxed for 2 h followed by addition of a solution of Zinc (II) acetate (0.92 g, 5 mmol) and sodium thiocyanate (0.406 g, 5 mmol) in a minimum amount of water. The mixture was refluxed for 2–3 h resulting in the formation of a white precipitate. More precipitate was obtained by removal of some solvent.


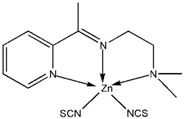


The product was collected by filtration, washed several times with ethanol until a white colored compound is obtained. It was re-crystallized from the same solvent (ethanol), filtered to remove the suspended impurities. Yield = 72%. Melting point 370–375 °C. Analysis Calculated: C, 35.27; H, 4.57; N, 11.22. Found: C, 35.17; H, 4.67; N, 11.21. IR (ATR cm^−1^) 2949.02 n(C-H), 2070.00 n(N=C=S), 1655.03 n(C=N), 1438.38 n(C-C), 1139.09 n(C-N), 477.06 n(M-N). UV-Vis [λ_max_ (nm) (DMSO)]. UV-Vis (DMSO) 497 (LMCT), 321 (n→π*); 233 (π→π*). ^1^H-NMR (DMSO-*d_6_*) 8.65, 8.64 [d, 1H, δ(Ar-H)pyr], 8.35–8.32 [m, 2H, δ(Ar-H)pyr], 7.95–7.94, 7.93 [m, 1H, δ(Ar-H)pyr], 3.79–3.77 [t, 2H, δ(N-CH2)], 2.77–2.75 [t, 2H, δ(CH_2_=)], 2.59 [s, 3H, δ(CH_3_)], 2.39 [s, 6H, δ(2CH_3_)]. ^13^C-NMR (DMSO-*d_6_*) 168.74 [1C, δ(C=N)], 147.99 δ(C), 147.16 δ(CH), 141.36 δ(CH), 135.03 δ(CH), 128.50 δ(CH) [5C, δ(Ar-pyr)], 56.35 [2C, δ(2CH_3_)], 44.55 [1C, δ(N-CH_2_)], 44.32 [1C, δ(CH_2_=)], 22.75 [δ(SCN)], 15.88 [1C, δ(CH_3_)].

*Aqua {2-morpholino-N-[1-(2-pyridyl)ethylidene] ethanamine N,N′,N′′}bis(thiocyanato) nickel(II) *(**10**): A mixture of 2-acetylpyridine (0.20 g, 1.65 mmol) and 4-(2-aminoethyl)morpholine(0.21 g, 1.65 mmol) in ethanol (20 mL) was refluxed for 2 h followed by addition of a solution of nickel(II) acetate tetrahydrate (0.41 g, 1.65 mmol) and sodium thiocyanate (0.134 g, 1.65 mmol) in a minimum amount of water. The mixture was refluxed for 2–3 h resulting in the formation of a greenish colored precipitate. More precipitate was obtained by removal of some solvent.


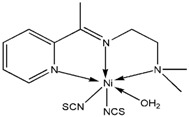


The product was collected by filtration, washed several times with ethanol until a green colored compound is obtained. It was re-crystallized from the same solvent (ethanol), filtered to remove the suspended impurities. Yield = 68%. Melting point 365–370 °C. Analytical Calculated: C, 42.27; H, 4.97; N, 16.43; S, 15.05. Found: C, 42.31; H, 4.98; N, 16.43; S, 15.09. IR (ATR cm^−1^) 2980.11 n(C-H), 2082.19 n(N=C=S), 1651.60 n(C=N), 1441.21 n(C-C), 1106.78 n(C-N), 659.16 n(OH_2_) 515.83 n(M-N), 461.51 n(M-O). UV-Vis (DMSO) 745.00 (d→d*); 574.00 (LMCT); 279.00 (π→π*).

### 3.1. X-Ray Structure Analyses

A single crystal of the complexes suitable for X-ray diffraction were mounted on a glass fibre using perfluoropolyether oil and cooled rapidly to 100 K in a stream of cold N_2_. Diffraction data were measured using a Bruker APEX II CCD area-detector diffractometer (graphite monochromated Mo Ka radiation, λ = 0.71073 Å). The orientation matrix, unit cell refinement and data reduction were all handled by the APEX II software (SAINT integration, SADABS absorption correction [47]. The structure was solved using direct methods and vectors in the program SHELXS-97 [48] and was refined by the full matrix least-squares method on F2 with SHELXL-97 [48]. All the nonhydrogen atoms were refined anisotropically and all the hydrogen atoms were placed at calculated positions and refined isotropically. Drawings of the molecules were produced with XSEED [49]. 

## 4. Pharmacology

### 4.1. Bacterial Strains

The antibacterial activities of the investigated compounds were tested against a panel of multi-drug resistant nosocomial bacterial pathogens, consisted of ten (10) isolates of methicillin-resistant *Staphylococcus aureus (MRSA)–MRSA 0804-25*, *MRSA 0805-21*, *MRSA 0806-1*, *MRSA 0807-1*, *MRSA 0807-7*, *MRSA 0807-19*, *MRSA 0808-35*, *MRSA 0809-15*, *MRSA 0809-24*, *MRSA 0809-38*; two (2) isolates of *Acinetobacter baumannii(AC)–AC 0612-7*, *AC 0903-21*, two (2) isolates of *Klebsiella pneumonia (KB)–KB 71*, *KB 83*, and two (2) isolates of *Pseudomonas aeruginosa (PA)–P. aeruginosa*. All tested bacterial strains of *MRSA* were of clinical origin and have shown resistance to at least four antibiotics (Table 6). For each bacterial species, six to ten strains were included in the antibacterial study to capture possible drug resistance variation within species. All bacterial strains used in this study were obtained from the bacteria culture collection of Biomedical Science Laboratory, Institute of Graduate Studies University of Malaya, Kuala Lumpur, Malaysia. 

#### 4.1.1. Antimicrobial Testing

The synthesized compounds were first screened for potential antibacterial activity by testing against two randomly selected strains from each bacterial species, using disc diffusion assay [50]. Briefly, a loopful of an overnight bacterial culture of each strain was suspended in sterile 0.85% saline (BDH) to the concentration of approximately 10^8^ cfu/mL (equivalent to 0.5 Mc Farland units) before inoculated evenly on the entire surface of Mueller Hinton agar (Oxoid) with a sterile cotton swab. Sterilized paper discs (Thermo Fisher, 6 mm diameter), impregnated with 30 µg of respective synthetic Schiff compound, and was placed on the inoculated agar plate. DMSO disc was used as a negative control in the test. The diameter of inhibition zone around the impregnated paper disc was measured after 18 h of incubation at 37 °C. All tests were performed in duplicate. Synthetic Schiff base compounds, in which inhibition zone was observed in any one of the four species tested, were selected for MIC and MBC determination with broth micro-dilution assay. Only compounds with MIC and MBC value lesser than “10 µg/mL” is considered potentially active against the corresponded bacterial species; and further tested against the entire collection of the corresponded bacterial species with disc diffusion assay. This was to determine the variation in the susceptibility responses to the tested compound within the bacterial species tested. The two selected strains for each species in the initial screening were: MRSA 0804-25 and MRSA 0807-7 for MRSA (Figure 3); AC 0612-7 and AC 0903-21 for *A. baumannii*; KB 71 and KB 83 for *K. pneumonia*; and PA45 and PA104 for *P. aeruginosa*. 

#### 4.1.2. MIC Determination

Minimum inhibition concentrations (MICs) were determined with a 96-well broth micro-dilution assay. The inoculum was prepared by suspending an overnight culture in a sterile 0.85% saline (BDH) to approximate 10^8^ cfu/mL (equivalent to 0.5 McFarland units) and further diluted in cation adjusted Mueller Hinton broth (CMHB; BD) to the concentration of 10^6^ cfu/mL. Two-fold serial dilutions of the tested compound were prepared in CMHB across the 96-well microtiter plate with the highest concentration starting from 750 ppm (750 µL/mL) in duplicate rows. The prepared suspension of the bacteria was added to each well except the negative control wells of the microtitre plate in a 1:1 ratio with final bacteria concentration of approximately “5 × 10^5^” cfu/mL. The inoculated microtitre plates were incubated at 37 °C for 24 h. The MIC was determined as the lowest concentration of the test compound that exhibits no visible growth. A further confirmative test was done by adding 30 µL of MTT dye (Sigma) to each well. Wells with color changes within 30–60 min incubation at room temperature were scored as positive growth. The lowest concentration of the tested compound that exhibits no color changes (yellow) is determined as the MIC value. 

#### 4.1.3. MBC Determination

The MBC, minimal bactericidal concentration was tested by sub culturing 100 µL from each well of the incubated plate into Mueller Hinton Agar. The MBC was determined as the lowest concentration of the test compound that exhibits no viable microorganism growth on the Mueller Hinton Agar plate. The antimicrobial compound is regarded as bactericidal if the MBC is not more than four times the MIC. 

## 5. Conclusions

In conclusion, Schiff-based complexes were highly inhibitive only against the Gram positive bacterium MRSA, and as such could be potential antibacterial drugs to control MRSA nosocomial infections in hospital settings. 

## Supplementary Data

CCDC 851793 and 851794 contain the supplementary crystallographic data for complex **8** and complex **9**. These data can be obtained free of charge via http://www.ccdc.cam.ac.uk/conts/retrieving.html, or from the Cambridge Crystallographic Data Centre, 12 Union Road, Cambridge CB2 1EZ, UK; fax: (+44) 1223-336-033; or Email: deposit@ccdc.cam.ac.uk.

## References

[1] Prakash A., Singh B.K., Bhojak N., Adhikari D. (2010). Synthesis and characterization of bioactive zinc(II) and cadmium(II) complexes with new Schiff bases derived from 4-nitrobenzaldehyde and acetophenone with ethylenediamine. Spectrochim. Acta.

[2] Sridhar S.K., Pandeya S.N., Stables J.P., Ramesh A. (2002). Anticonvulsant activity of hydrazones, Schiff and Mannich bases of Isatin derivatives. Eur. J. Pharm. Sci..

[3] Bharti S.K., Nath G., Tilak R., Singh S.K. (2010). Synthesis, anti-bacterial and anti-fungal activities of some novel Schiff bases containing 2,4-disubstituted thiazole ring. Eur. J. Med. Chem..

[4] Kalagouda B.G., Manjula S.P., Ramesh S.V., Rashmi V.S., Siddappa A.P. (2006). X-ray crystal structure of the *N*-(2-hydroxy-1-naphthalidene)phenylglycineSchiff base. Synthesis and characterization of its transition metal complexes. Trans. Met. Chem..

[5] Cukurovali A., Yilmaz İ., Kirbag S. (2006). Spectroscopic characterization and biological activity of salicylaldehyde thiazolyl hydrazone ligands and their metal complexes. Trans. Met. Chem..

[6] Tansir A., Nahid N., Shadma P. (2008). Synthesis, characterization and antimicrobial studies of newly developed polymeric schiff base and its metal-polychelates. J. Coord. Chem..

[7] Manabu F., Hisanobu W., Takayuki M., Toshiyuki S. (1990). Preparation of 14-, 18-, and 22-membered tetraaza macrocycles and their complexing ability for copper(ii) and nickel(II) ions. Bull. Chem. Soc. Jpn..

[8] Pandeya S.N., Sriram D., Nath G., DeClercq E. (1999). Synthesis, antibacterial, antifungal and anti HIV activities of Schiff and Mannich bases derived from isatin derivatives and N-[4-(4-chlorophenyl) thiazol-2-yl] thiosemicarbazide. Eur. J. Pharm. Sci..

[9] Zhang J.A., Pan M., Zhang J.Y., Kang B.S., Su C.Y. (2009). Syntheses, structures and bioactivities of cadmium(II) complexes with a tridentate heterocyclic N- and S-ligand. Inorg. Chim. Acta.

[10] Mandal S., Karmakar T.K., Ghosh A., Fleck M., Bandyopadhyay D. (2011). Synthesis, crystal structure and antibacterial activity of a group of mononuclear manganese(II) Schiff base complexes. Polyhedron.

[11] Yusnita J., Puvaneswary S., Ali H.M., Robinson W.T., Lin T.K. (2009). Synthesis, structural characterization and antibacterial activity of 2,6-diacetylpyridine bis(benzenesulfonohydrazide) Schiff bases and their copper(II) complexes. Polyhedron.

[12] Pignatello R., Panico A., Mazzone P., Pinizzotto M.R., Garozzo A., Fumeri P.M. (1994). Schiff bases of *N*-hydroxy-*N*′-aminoguanidines as antiviral, antibacterial and anticancer agents. Eur. J. Med. Chem..

[13] Ceyhan G., Çelik C. (2011). Antioxidant, electrochemical, thermal, antimicrobial and alkane oxidation properties of tridentate Schiff base ligands and their metal complexes. Spectrochim. Acta.

[14] Tajudeen S.S., Radha E. (2009). Synthesis, characterization and antimicrobial activity of transition metal complexes of schiff base derivatives from isonicotinic acid hydrazide. Asian J. Chem..

[15] Bagihalli G.B., Avaji P.G. (2008). Synthesis, spectral characterization, in vitro antibacterial, antifungal and cytotoxic activities of Co(II), Ni(II) and Cu(II) complexes with 1,2,4-triazole Schiff bases. Eur. J. Med. Chem..

[16] Ispir E., Toroglu S., Kayraldrz A. (2008). Syntheses, characterization, antimicrobial and genotoxic activities of new Schiff bases and their complexes. Transit. Met Chem..

[17] Guidos R.J. (2010). The 10×'20 Initiative: Pursuing a Global Commitment to Develop 10 New Antibacterial Drugs by 2020. Am. Clin. Infect. Dis..

[18] Esposito S., Leone S. (2007). Antimicrobial treatment for Intensive Care Unit (ICU) infections including the role of the infectious disease specialist. Int. J. Antimicrob. Agents.

[19] Noskin G.A., Siddiqui F., Stosor V., Hacek D., Peterson L.R. (1999). In vitro activity of linezolid against important Gram-positive bacteria pathogens including vancomycin-resistant enterococci. Antimicrob. Agent Chemother..

[20] Prystowsky J., Siddiqui F., Chosay J., Shinabarger D.L., Millichap J., Peterson L.R., Noskin G.A. (2001). Resistance to linezolid: Characterization of mutations in rRNA and comparison of their occurrences in vancomycin-resistant enterococci. Antimicrob. Agent Chemother..

[21] Raparti V., Chitre T., Bothara K.G., Kumar V., Dangre S., Khachane C., Gore S., Deshmane B. (2009). Novel 4-(morpholin-4-yl)-N′-(arylidene)benzohydrazides: Synthesis, antimycobacterial activity and QSAR invest- tiga-tions. Eur. J. Med. Chem..

[22] Laskar I.R., Maji T.K., Das D., Lu T.H., Wong W.-T., Okamoto K.-i., Chaudhuri N.R.  (2001). Syntheses, characterisation and solid state thermal studies of 1-(2-aminoethyl)piperidine (L), 1-(2-aminoethyl)pyrrolidine (L) and 4-(2-aminoethyl)morpholine (L.) complexes of nickel(II): X-ray single crystal structure analyses of trans-[NiL2(CH3CN)2](ClO4)2, trans-[NiL2(NCS)2] and trans-[NiL-2(NCS)2]. Polyhedron.

[23] Raman N., Selvan A., Sudharsan S. (2011). Metallation of ethylenediamine based Schiff base with biologically active Cu(II), Ni(II) and Zn(II) ions: Synthesis, spectroscopic characterization, electrochemical behaviour, DNA binding, photonuclease activity and in vitro antimicrobial efficacy. Spectrochim. Acta A.

[24] Khan N.U., Pandya N., Prathap K.J., Kureshy R.I., Abdi S.H., Mishra S., Bajaj H.C. (2011). Chiral discrimination asserted by enantiomers of Ni (II), Cu (II) and Zn (II) Schiff base complexes in DNA binding, antioxidant and antibacterial activities. Spectrochim. Acta.

[25] Nakamoto K. (1978). Infrared and Raman Spectra of Inorganic and Coordination Compounds.

[26] Bhowmik P., Chattopadhyyay S., Drew M.G.B., Diaz C., Ghosh A. (2010). Synthesis, structure and magnetic properties of mono- and di-nuclear nickel(II) thiocyanate complexes with tridentate N3 donor Schiff bases. Polyhedron.

[27] Banerjee S., Wu B., Lassahn P.G., Janiak C., Ghosh A. (2005). Synthesis, structure and bonding of cadmium(II) thiocyanate systems involving nitrogen containing ligands of different denticity. Inorg. Chim. Acta.

[28] Chattopadhyay S., Drew M.G.B., Ghosh A. (2005). Binuclear complexes of M(II) thiocyanate (M = Ni and Cu) containing a tridentate Schiff base ligand: synthesis, structural diversity and magnetic properties. Eur. J. Inorg. Chem..

[29] Mohamed M., Hapipah M.A., Mahmood A.A., Robinson T.W. (2009). Synthesis, structural characterization, and anti-ulcerogenic activity of Schiff base ligands derived from tryptamine and 5-chloro, 5-nitro, 3,5-ditertiarybutyl salicylaldehyde and their nickel(II), copper(II), and zinc(II) complexes. Polyhedron.

[30] Lakshmi B., Avaji P.G., Shivananda K.N., Naggella P., Manohar S.H., Mahendra K.N. (2011). Synthesis, spectral characterization and in vitro microbiological evaluation of novel glyoxal, biacetyl and benzil bis-hydrazone macrocyclic Schiff bases and their Co(II), Ni(II) and Cu(II) complexs. Polyhedron.

[31] Deoghoria S., Mostafa G., Lu T.H., Chandra S.K. (2004). Synthesis, characterisation and properties of manganese(II) complexes having pseudohalide coordination: X-ray crystal structure of an unusually distorted hexacoordinated [MnL(NCS)](ClO_4_) species (L = pentadentate Schiff base ligand). Ind. J. Chem..

[32] Shahabadi N., Kashanian S. (2010). DNA binding and DNA cleavage studies of a water soluble cobalt(II) complex containing dinitrogen Schiff base ligand: The effect of metal on the mode of binding. Eur. J. Med. Chem..

[33] Chen W., Li Y., Cui Y., Zhang X. (2010). Synthesis, molecular docking and biological evaluation of Schiff base transition metal complexes as potential urease inhibitors. Eur. J. Med. Chem..

[34] Creaven B.S., Duff B., Egan D.A., Kavanagh K., Rosair G., Thangella V.R., Walsh M. (2010). Anticancer and antifungal activity of copper(II) complexes of quinolin-2(1H)-one-derived Schiff bases. Inorg. Chim. Acta.

[35] Raman N., Jeyamurugan R., Senthilkumar R., Rajkapoor B., Franzblau S.G. (2010). In vivo and in vitro evaluation of highly specific thiolate carrier group copper(II) and zinc(II) complexes on Ehrlich ascites carcinoma tumor model. Eur. J. Med. Chem..

[36] Ali M.A., Mirza A.H., Tan A.L., Bujang F.H., Hamid M.H.S.A., Bernhardt P.V. (2008). Preparation and structural characterization of nickel(II), cobalt(II), zinc(II) and tin(IV) complexes of the isatin Schiff bases of S-methyl and S-benzyldithiocarbazates. Polyhedron.

[37] Ceyhan G., Çelik C., Uruş S., Demirtaş İ., Elmastaş M., Tümer M. (2011). Antioxidant, electrochemical, thermal, antimicrobial and alkane oxidation properties of tridentate Schiff base ligands and their metal complexes. Spectrochim. Acta A Mol. Biomol. Spectr..

[38] Hisham N.A.I., Gwaram N.S., Khaledi H., Ali H.M. (2011). Dichlorido{2-morpholino-N-[1-(2-pyridyl)ethylidene] ethanamine- 3N,N',N''}zinc(II). Acta Crystallogr..

[39] Addison A.W., Rao T.N., Reedijk J., Rijn V.J., Verschoor G.C. (1984). Synthesis, structure, and spectroscopic properties of copper(II) compounds containing nitrogen-sulphur donor ligands; the crystal and molecular structure of aqua[1,7-bis(*N*-methylbenzimidazol-2′-yl)-2,6-dithiaheptane]copper(II) perchlorate. J. Chem. Soc. Dalton Trans..

[40] You Z.L., Chi J.Y. (2006). Synthesis, crystal structures and antibacterial activities of two Schiff base zinc(II) complexes. Synth. React. Inorg. Met.-Org., Nano-Met. Chem..

[41] Zakrzewski G., Lingafelter E.C. (1970). The crystal and molecular structure of dibromo-1- (2-pyridyl)-2,5-diaza-5-methyl-hexa-1-enezinc(II). Inorg. Chim. Acta.

[42] Cai B.H. (2009). (2-Morpholinoeth-yl)(2-pyridylmethyl-ene)amine]dithio-cyanato-zinc(II). Acta Crystallgr..

[43] Chen G., Bai Z.P., Qu S.J. (2005). *N*,*N*'-Dimethyl-*N*''-(2-pyridylmethylene)ethane-1,2-diamine] dithiocyanato zinc(II). Acta Crystallogr..

[44] Gwaram N.S., Khaledi H., Ali H.M. (2011). *catena*-Poly-*N*,*N*-dimethyl-*N*'-[1-(pyridin-2-yl)ethylidene]ethane-1,2-diamine-κ^3^*N*,*N*',*N*''}(thiocyanato-κ*N*)cadmium]-μ-thiocyanato-κ^2^*S*:*N*. Acta Crystallogr..

[45] Chohan Z.H., Arif M., Akhtar M.A., Supuran C.T. (2006). Metal-based antibacterial and antifungal agents: Synthesis, characterization, and in vitro biological evaluation of Co(II), Cu(II), Ni(II), and Zn(II) complexes with amino acid-derived compounds. Bioinorg. Chem. Appl..

[46] Chohan Z.H., Scozzafava A., Supuran C.T. (2003). Zinc complexes of benzothiazole-derived Schiff bases with antibacterial activity. J. Enzyme Inhib. Med. Chem..

[47] (2007). Bruker APEX2 and SAINT.

[48] Sheldrick G.M. (2008). A short history of SHELX. Acta Crystallogr. A.

[49] Barbour L.J. (2001). X-Seed-A software tool for supramolecular crystallography. J. Supramol. Chem..

[50] Clinical and Laboratory Standards Institute (CLSI) (2011). Performance Standard for Antimicrobial Susceptibility Testing; Twenty-First Information Supplement; CLSI document M100-S21.

